# Broadening the Action Spectrum of TiO_2_-Based Photocatalysts to Visible Region by Substituting Platinum with Copper

**DOI:** 10.3390/nano12091584

**Published:** 2022-05-07

**Authors:** Andrey A. Saraev, Anna Yu. Kurenkova, Evgeny Yu. Gerasimov, Ekaterina A. Kozlova

**Affiliations:** Federal Research Center, Boreskov Institute of Catalysis SB RAS, Lavrentieva Ave. 5, 630090 Novosibirsk, Russia; asaraev@catalysis.ru (A.A.S.); kurenkova@catalysis.ru (A.Y.K.); gerasimov@catalysis.ru (E.Y.G.)

**Keywords:** photocatalysis, CO_2_ reduction, TiO_2_, CH_4_ production, visible light, ultraviolet light, copper oxides, platinum

## Abstract

In this study, TiO_2_-based photocatalysts modified with Pt and Cu/CuO_x_ were synthesized and studied in the photocatalytic reduction of CO_2_. The morphology and chemical states of synthesized photocatalysts were studied using UV-Vis diffuse reflectance spectroscopy, high-resolution transmission electron microscopy, and X-ray photoelectron spectroscopy. A series of light-emitting diodes (LEDs) with maximum intensity in the range of 365–450 nm was used to determine the action spectrum of photocatalysts. It is shown for, the first time, that the pre-calcination of TiO_2_ at 700 °C and the use of Cu/CuO_x_ instead of Pt allow one to design a highly efficient photocatalyst for CO_2_ transformation shifting the working range to the visible light (425 nm). Cu/CuO_x_/TiO_2_ (calcined at 700 °C) shows a rate of CH_4_ formation of 1.2 ± 0.1 µmol h^−1^ g^−1^ and an overall CO_2_ reduction rate of 11 ± 1 µmol h^−1^ g^−1^ (at 425 nm).

## 1. Introduction

Today, humankind faces the problem of an increase in the emission of greenhouse gases, and their utilization is increasingly becoming the actual goal. Additionally, special attention is paid to utilizing the major component of greenhouse gases—CO_2_ [1,2]. A promising approach to utilizing CO_2_ is its photocatalytic conversion to more reactive substances, such as CO and CH_4_. This technology allows reducing the amount of CO_2_ in the atmosphere [3]. Moreover, the products of this process are organic compounds, such as methane, methanol, ethanol, and formaldehyde, which could be then converted into valuable organic components or fuels [4]. It is worth noting that such organic compounds could be synthesized using renewable sources: light, CO_2_, and water [5].

The thermodynamic stability and kinetic inertia of CO_2_ limit its use in the chemical industry. The development of technologies that allow the conversion of CO_2_ into useful organic compounds under “soft” conditions will increase the appeal of this process and facilitate its large-scale implementation. Exposure to visible or UV light irradiation is known to remove thermodynamic limitations on the reductive conversion of carbon dioxide in the presence of water into organic compounds, which makes the process of photocatalytic CO_2_ reduction one of the most promising technologies, but the large-scale implementation of this process is hampered by the lack of effective photocatalysts.

Metal oxides, especially TiO_2_, are widely used as photocatalysts because of their stability and their ability to generate the electron–hole pairs under light irradiation and at a low cost [6,7]. Modification with metals allows one to enhance the activity of titania due to the separation of photogenerated charge carriers (electron–hole pairs) [8,9]. Currently, platinum is the most used metal as a cocatalyst, as it has a high working function of the electron (5.6 eV) and, therefore, promotes effective charge separation [10,11]. Despite the high activity of materials including Pt as cocatalyst, there is a necessity to develop an effective photocatalyst with non-noble metal from a practical point of view. One of the promising cocatalysts is Cu and its oxides, both Cu_2_O and CuO have narrow bandgaps and are able to absorb visible light [12,13,14,15,16]. It should be noted that calcination of TiO_2_ at high temperature leads to a shift in adsorption edge to a longer wavelength [17]. Such thermal activation should lead to enhanced photocatalytic activity under visible light irradiation. The advantage of this method is its simplicity compared with other methods for shifting the TiO_2_ absorption edge, such as reconstructing the TiO_2_ surface, embedding quantum dots, creating defects, or doping TiO_2_ lattice [18,19,20,21,22].

At the same time, although research on this process has been ongoing for the past 20 years, there is still no consensus on which photocatalysts are the most active, how the reduction of CO_2_ occurs, and what affects the distribution of reduction products. Most studies are aimed at the synthesis of active material for photocatalytic reduction of CO_2_ [3], while the identification of regularities between the nature of the products formed, as well as the rate of CO_2_ reduction and the state of the catalyst, facilitate the formulation of a scientific basis for creating an efficient photocatalytic system. In the case of using Cu as a cocatalyst, special attention should be paid to studying the photocatalyst surface due to the chemical versatility of Cu and the possible formation of copper oxides [23].

This research focused on a detailed study of the products of CO_2_ reduction that depend on the nature of surface TiO_2_-based photocatalyst using Cu and Pt as cocatalysts. To explain the mechanism of the photocatalyst operation, the reduction of CO_2_ was studied under irradiation of light-emitting diodes with different wavelengths in the range from 365 to 450 nm, and the catalysts were characterized by transmission electron microscopy, X-ray photoelectron spectroscopy, and UV-Vis spectroscopy. In the current manuscript, the influence of Pt and Cu/CuO_x_, the thermal activation of TiO_2_, and the light source wavelength on the product distribution and photocatalyst activity in CO_2_ reduction are defined under the same conditions, for the first time. The article reveals the characteristics of the photocatalytic system that contribute to the predominant formation of CH_4_ during photocatalytic CO_2_ reduction. A simple approach involving heat treatment of TiO_2_ and Cu/CuO_x_ deposition is proposed to synthesize visible light active photocatalyst.

## 2. Materials and Methods

### 2.1. Photocatalyst Synthesis

Commercial titanium dioxide Evonik P25 (Evonik Industries AG, Essen, Germany) was used for photocatalyst preparation. Cocatalysts deposition was carried out by impregnation method using H_2_PtCl_6_ (Reakhim, Moscow, Russia, 98%), Cu(NO_3_)_2_·2.5H_2_O (Reakhim, Moscow, Russia, 98%), and NaBH_4_ (Acros Organics, Geel, Belgium, 99%) aqueous solutions. For thermal activation, TiO_2_ was pre-calcined at 700 °C for 3 h. This sample is marked as “TiO_2_ 700” in subsequent sections. Platinum and copper were deposited from aqueous solutions of H_2_PtCl_6_ and Cu(NO_3_)_2_ on the surface of unmodified TiO_2_ and TiO_2_ calcined at 700 °C. The synthesis is described in detail elsewhere [24,25]. The calculated metal loading was 1 wt.% and 5 wt.% for Pt and Cu, respectively.

### 2.2. Photocatalyst Characterization

The synthesized catalysts were characterized by UV-Vis diffuse reflectance spectroscopy, high-resolution transmission electron microscopy (HR TEM), inductively coupled plasma atomic emission spectroscopy, and X-ray photoelectron spectroscopy (XPS).

The diffuse reflectance UV-Vis spectra were measured using a UV-2501 PC spectrophotometer with an ISR-240A diffuse reflectance unit (Shimadzu, Kyoto, Japan).

The structure and microstructure of the photocatalysts were studied with HR TEM using a ThemisZ electron microscope (Thermo Fisher Scientific, Waltham, MA, USA) operated at an accelerating voltage of 200 kV. The microscope is equipped with a corrector of spherical aberrations, which provided a maximum lattice resolution of 0.06 nm, and a SuperX energy-dispersive spectrometer (Thermo Fisher Scientific). Images were recorded using a Ceta 16 CCD sensor (Thermo Fisher Scientific). For electron microscopy studies, samples were deposited on perforated carbon substrates attached to aluminum grids using an ultrasonic dispersant.

Inductively coupled plasma atomic emission spectroscopy (ICP-AES) was used to perform elemental analysis of the synthesized photocatalysts. ICP-AES was carried out on an Optima 4300 5DV spectrometer (PerkinElmer Inc., Waltham, MA, USA).

The relative surface concentration of elements and their chemical states were investigated using XPS. The study was carried out using an X-ray photoelectron spectrometer (SPECS Surface Nano Analysis GmbH, Berlin, Germany), equipped with an XR-50 X-ray source and PHOIBOS-150 hemispherical electron energy analyzer. The Cu*2p*, Pt*4f*, Ti*2p*, and O*1s* XPS core-level spectra were recorded under ultrahigh vacuum conditions. The core-level spectra were obtained using the non-monochromatic Al Kα radiation (hυ = 1486.61 eV). The charge correction was performed by setting the Ti*2p_3/2_* peak at 459.0 eV, which corresponded to titanium in the Ti^4+^ state of the TiO_2_ lattice. Relative concentrations of elements were determined from the integrated intensities of the core-level spectra using their cross-sections according to Scofield [26]. For detailed analysis, the spectra were fitted into several peaks after background subtraction using the Shirley method. The fitting procedure was performed using the CasaXPS software [27]. The line shape of the peaks was approximated by the multiplication of the Gaussian and Lorentzian functions.

### 2.3. Photocatalytic Activity Measurements

**Figure 1:**
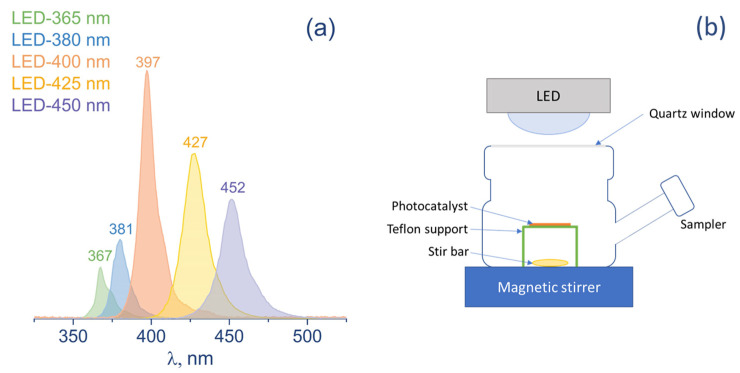
(**a**) Spectra of LEDs with the wavelength at maximum intensity; (**b**) scheme of reactor for photocatalytic study.

The reduction of CO_2_ was carried out in a batch reactor (70 mL) with a quartz window (16 cm^2^) under light irradiation (Figure 1). A suspension consisting of a catalyst (30 mg) and water (700 µL) was deposited on a glass support (8 cm^2^) and dried at 50 °C in an air atmosphere. The resulting thickness of catalyst coverage is about 100 µm. After the deposition, the catalyst was irradiated by UV light (380 nm) for 30 min to remove organic contaminations from the photocatalyst surface and then installed in the reactor containing 1 mL of deionized water. Pure water without any additional sacrificial agents was used in all photocatalytic experiments. After that, the reactor was purged with CO_2_ (>99.94% purity) for 1 h. A number of light emission diodes (LEDs) with wavelength and surface power density of 365 (10 mW/cm^2^), 380 (17 mW/cm^2^), 400 (60 mW/cm^2^), 425 (56 mW/cm^2^), and 450 (42 mW/cm^2^) nm was used as a light source. The surface power density was measured at the sample position. 

A kinetic experiment was carried out for 24 h. Gas probe was taken using a gas syringe (500 µL) and analyzed with a gas chromatograph “GH-1000” (Chromos, Moscow, Russia) equipped with the flame ionization detector and thermal conductivity detector to identify the products of CO_2_ reduction and H_2_, respectively.

The total rate of photocatalytic CO_2_ reduction was calculated according to the following equation:

(1) $$W\;({\mathrm{CO}}_{2})=\frac{8n\left({\mathrm{CH}}_{4}\right)+2n\left(\mathrm{CO}\right)}{t\;m}$$

where *n*(CH_4_) and *n*(CO) are the amounts of CH_4_ and CO (µmol), 2 and 8 are the coefficients for electron balance, *t* is the time of reaction (h), and *m* is the weight of photocatalyst (g).

The selectivity of CH_4_ formation was defined as

(2) $$S\;({\mathrm{CH}}_{4})=\frac{8\;n\left({\mathrm{CH}}_{4}\right)}{t\;m\;W\left({\mathrm{CO}}_{2}\right)}\times 100\%$$

The apparent quantum efficiency (AQE) was calculated according to the following equation:

(3) $$\mathrm{AQE}=\frac{W\left({\mathrm{CO}}_{2}\right)}{N_{\mathrm{phot}}}\times 100\%$$

where N_phot_ is the calculated photon flux equal to 5.8 mmol h^−1^ for LED-425.

## 3. Results and Discussion

### 3.1. Photocatalyst Characterization

**Figure 2:**
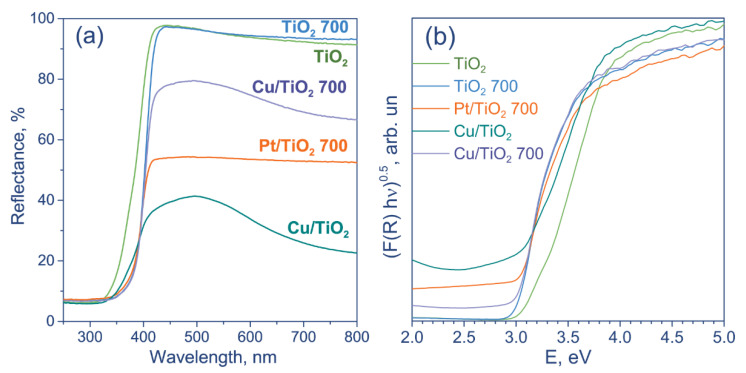
(**a**) Diffuse reflectance spectra of photocatalysts under study; (**b**) Tauc plots.

The optical properties of the synthesized photocatalysts were studied by UV-Vis diffuse reflectance spectroscopy (Figure 2a). The non-modified titania has a single, steep absorption edge around 380 nm, which is typical for Evonik (Degussa) P25. After calcination, the TiO_2_ 700 sample demonstrates a redshift, and the absorption edge is about 400 nm. Evonik P25 is known to consist of two TiO_2_ phases with different bandgap energy: anatase (3.2 eV) and rutile (3.0 eV) [28,29]. The observed effect indicates a structural change in TiO_2_ during calcination and an increase in rutile content for TiO_2_ 700. Calculated bandgap energies using Tauc plots are 3.14 eV for unmodified TiO_2_ and 3.02 eV for TiO_2_ 700 (Figure 2b). Earlier, it has been shown that the calcination of TiO_2_ at 700 °C changes anatase to rutile ratio from 85:15 to 22:78 [24], which is in agreement with the UV-Vis spectroscopy study of the synthesized catalysts.

The actual metal content established by ICP-AES was around 0.7% for platinized samples and 4.5% for Cu-containing catalysts.

The deposition of platinum and copper promotes the adsorption of visible light for both TiO_2_ and TiO_2_ 700. Platinum was shown to possess a localized surface plasmon resonance in the visible region [30]. In the case of Cu-containing samples, the increased absorption is caused by the intrinsic adsorption of copper oxides due to the narrow bandgap width of CuO (1.7 eV) and Cu_2_O (2.1 eV) [31,32]. The difference in the reflectance between Cu/TiO_2_ and Cu/TiO_2_ 700 photocatalysts may be due to the different content of CuO_x_ and Cu since oxides enhance the light absorption of the samples, whereas metal Cu does not [33]. Adsorption beyond 600 nm is related to the bandgap transitions of CuO [12]. We speculated that the content of CuO in Cu/TiO_2_ is higher than in Cu/TiO_2_ 700, which was further confirmed by HR TEM and XPS.

### 3.2. Kinetic Experiments

The activity of the synthesized photocatalysts was studied in the reduction of CO_2_ using a series of LEDs with an intensity maximum at about 365, 380, 400, 425, and 450 nm as light sources to define the spectrum of action of photocatalysts.

Since H_2_O was added to the studied system, the following processes could proceed on the photocatalyst surface [8,34]:

CO_2_ + 2H^+^ + 2e^−^ = CO + H_2_O, E^0^ = −0.53 eV vs. NHE at pH 7 (4)

CO_2_ + 8H^+^ + 8e^−^ = CH_4_ + 2H_2_O, E^0^ = −0.24 eV vs. NHE at pH 7 (5)

2H^+^ + 2e^−^ = H_2_, E^0^ = −0.41 eV vs. NHE at pH 7 (6)

The products of reactions were analyzed using gas chromatography: CO and CH_4_ were the main products, and H_2_ was detected in negligible amount—less than 0.05 µmol after 24 h of reaction for the most active catalyst, i.e., Cu/TiO_2_.

**Figure 3:**
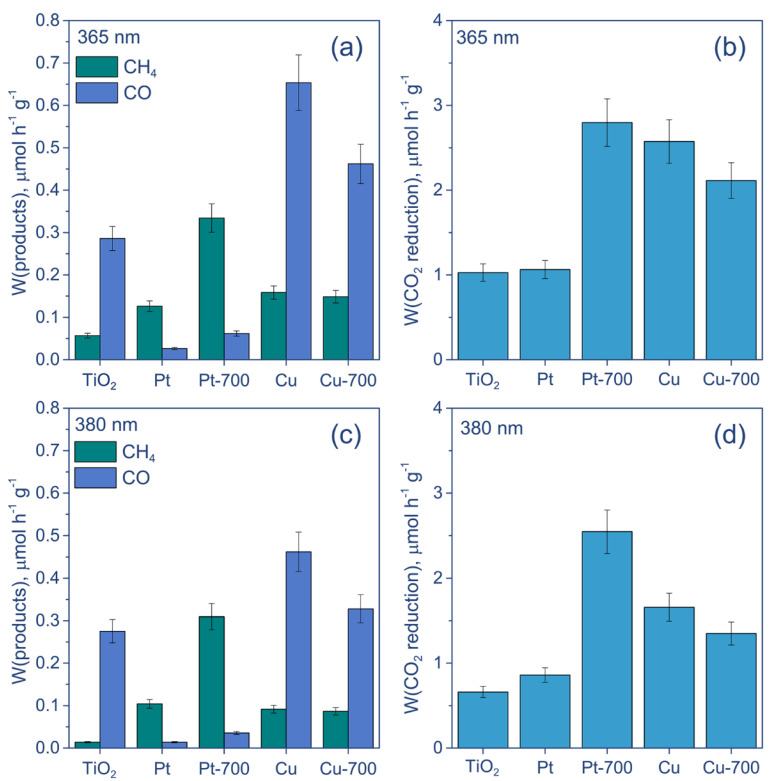
The results of activity study of photocatalytic reduction of CO_2_ under irradiation by light of (**a**,**b**) LED-365 nm and (**c**,**d**) LED-380 nm. Conditions: m(cat.) = 30 mg, P_0_(CO_2_) = 1 atm, t = 24 h. Designation: “Pt” and “Cu” means Pt/TiO_2_ and Cu/TiO_2_, respectively; “Pt-700” and “Cu-700” means Pt/TiO_2_ 700 and Cu/TiO_2_ 700, respectively.

The rates of CO, CH_4_ formation, and total CO_2_ reduction are presented in Figure 3. One can see that the Pt deposition changes the distribution of products and greatly increases the amount of CH_4_. The Pt/TiO_2_ and Pt/TiO_2_ 700 samples predominantly reduce CO_2_ to CH_4_, and only a little amount of CO is detected, except for the LED-450 nm. When using Cu-containing samples, the dependence is more complex. Under UV irradiation, the formation rates of CO are higher than those of CH_4_. Nevertheless, the amounts of CH_4_ produced over Cu/TiO_2_ and Cu/TiO_2_ 700 are similar to those for Pt/TiO_2_. At the same time, Cu/TiO_2_ and Cu/TiO_2_ 700 yield CO at significantly higher rates than Pt/TiO_2_, resulting in a higher overall CO_2_ reduction efficiency. The activity of these photocatalysts is close to that of Pt/TiO_2_ 700 under LED-365 nm, which demonstrates that the highest rate of overall CO_2_ reduction under UV light is equal to 2.8 ± 0.3 µmol h^−1^ g^−1^.

According to UV-Vis spectra, all photocatalysts exhibit strong light absorption at a wavelength of 365 nm. However, with an increase in the wavelength to 380 nm, the absorption of the samples obtained without preliminary calcination of TiO_2_ decreases compared with the calcined samples. Pt/TiO_2_ 700 demonstrates higher activity under LED-380 nm and LED-365 nm, compared with Pt/TiO_2_. Therefore, the difference in activity between Pt/TiO_2_ and Pt/TiO_2_ 700 is not caused only by different light absorption. The most likely reason for the improved activity under UV light is the increased lifetime of photogenerated charge carriers—electrons and holes—for Pt/TiO_2_ 700 due to the efficient transfer of photogenerated electrons from TiO_2_ to metal particles. To clarify the reason for the increased activity of the Pt/TiO_2_ 700 sample under irradiation with LED-365 nm, compared with Pt/TiO_2_, the photocatalysts were characterized using HR TEM and XPS.

**Figure 4:**
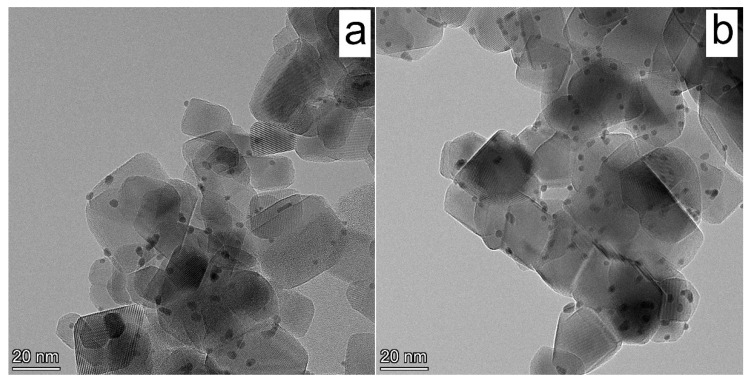
HR TEM micrographs of (**a**) fresh Pt/TiO_2_ and (**b**) Pt/TiO_2_ 700 photocatalysts.

The HR TEM micrographs of the Pt/TiO_2_ and Pt/TiO_2_ 700 are presented in Figure 4. For Pt/TiO_2_, one can identify well-crystallized TiO_2_ nanoparticles with a size of 10–25 nm (Figure 4a), whereas in the case of Pt/TiO_2_ 700 catalyst (Figure 4b), the average size of crystallized TiO_2_ nanoparticles increases to about 35–45 nm. The effect of thermal treatment has been previously studied and shown to lead to a decrease in the specific surface area of the TiO_2_ support, an increase in crystallite size, and the part of rutile [24]. It should be noted that, for both supports, the platinum nanoparticles have a narrow particle size distribution, with mean particle sizes of 1.9 and 2.2 nm for Pt/TiO_2_ and Pt/TiO_2_ 700, respectively. The difference between these catalysts is the aggregates of Pt nanoparticles observed in the case of Pt/TiO_2_. For Pt/TiO_2_ 700, Pt nanoparticles are well distributed on the TiO_2_ surface and do not form agglomerate structures.

The XPS study of the Pt/TiO_2_ and Pt/TiO_2_ 700 samples revealed that the [Pt]/[Ti] ratio in the Pt/TiO_2_ 700 photocatalyst increases by a factor of two, compared with Pt/TiO_2_ (Table 1). This effect is caused by a decrease in the surface area of TiO_2_, as shown in our previous research [24]. Since the thermal treatment of TiO_2_ was carried out in the same manner, we assumed a similar change in surface area. The binding energy of the Pt*4f_7/2_* peak is 70.9 eV for both catalysts, which corresponds to platinum in the metallic state (Figure S1a). Additionally, these samples were studied using XPS after a photocatalytic reaction under LED-400 nm, and no change in the state of Pt was observed (Figure S1b). The [O]/[Ti] ratio after calcination of the initial TiO_2_ sample and after carrying out the photocatalytic reaction almost does not change. This small increase could be related to the formation of cation vacancies in a negligible amount [35]. Thus, the higher activity of Pt/TiO_2_ 700 is due to the high surface concentration of platinum nanoparticles and enhanced absorption properties of calcinated TiO_2_.

**Figure 5:**
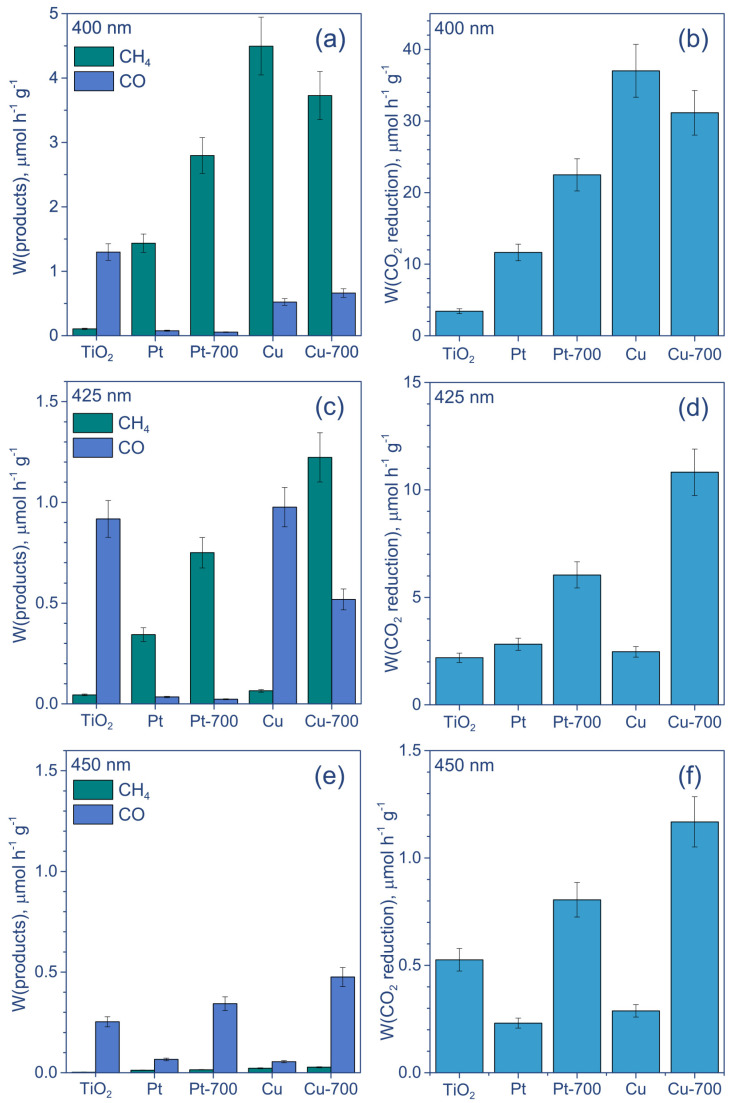
The results of activity study of photocatalytic reduction of CO_2_ under irradiation by light of (**a**,**b**) LED-400 nm, (**c**,**d**) LED-425 nm, and (**e**,**f**) LED-450 nm. Conditions: m(cat.) = 30 mg, P_0_(CO_2_) = 1 atm, t = 24 h. Designation: “Pt” and “Cu” means Pt/TiO_2_ and Cu/TiO_2_, respectively; “Pt-700” and “Cu-700” means Pt/TiO_2_ 700 and Cu/TiO_2_ 700, respectively.

Under light irradiation with a maximum intensity at 400 nm, both Cu-containing photocatalysts demonstrate higher CH_4_ formation rates than CO (Figure 5a). Moreover, their activities are much higher than that of both platinized samples and achieve 37 ± 4 and 31 ± 3 µmol h^−1^ g^−1^ for Cu/TiO_2_ and Cu/TiO_2_ 700, respectively (Figure 5b). When using LED-425 nm, the activity of photocatalysts based on calcinated TiO_2_ (Pt/TiO_2_ 700 and Cu/TiO_2_ 700) exceeds the activity of photocatalysts based on uncalcined TiO_2_ (Figure 5c,d). Additionally, Cu/TiO_2_ produces more CO than CH_4_, while Cu/TiO_2_ 700 is still more active toward CH_4_ formation. Finally, the use of LED-450 nm leads to the predominant formation of CO for all studied catalysts, but the efficiency of this process is low (Figure 5e,f). Nevertheless, calcined photocatalysts are still more active in overall CO_2_ reduction, with a reaction rate of about 1 µmol h^−1^ g^−1^.

The calcinated samples have a shifted adsorption edge, compared with unmodified ones and, therefore, enhanced absorption and improved photocatalytic activity under visible light (425 and 450 nm). Since the dependence under LED-400 nm is more complex, the Cu-containing samples were studied using XPS and HR TEM in the high-angle annular dark-field scanning mode (HAADF STEM). The HAADF STEM allows one to make micrographs of samples with high contrast, with the bright area corresponding to the atoms with higher Z.

**Figure 6:**
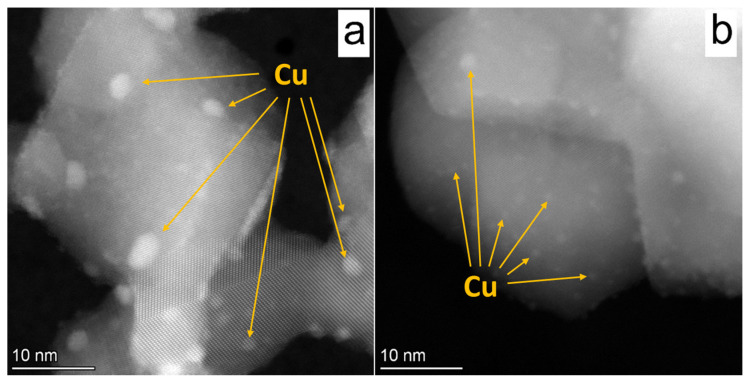
HAADF STEM micrographs of (**a**) fresh Cu/TiO_2_ and (**b**) Cu/TiO_2_ 700 photocatalysts. The arrows indicate the copper nanoparticles. The bright area corresponds to copper atoms.

The HAADF STEM micrographs of the Cu/TiO_2_ and Cu/TiO_2_ 700 are presented in Figure 6. For Cu/TiO_2_, one can see that copper nanoparticles have a wide particle size distribution in the range of 1.5–5 nm, whereas, in the case of Cu/TiO_2_ 700, a narrow particle size distribution in the range of 1–2 nm is found. It is worth noting that, in the case of Cu/TiO_2_ 700, the agglomerates of Cu nanoparticles are not observed. Moreover, in the case of Cu/TiO_2_, the EDX mapping reveals the presence of massive Cu particles of 200–400 nm (Figure S2a). Thus, the use of calcined TiO_2_ as support allows the synthesis of a photocatalyst with a fine size distribution of Cu nanoparticles. 

According to the results of UV-Vis spectroscopy, Cu/TiO_2_ and Cu/TiO_2_ 700 absorb visible light to varying degrees, but light absorption in this region indicates the presence of copper oxides in both samples. This assumption was clearly confirmed by the XPS study (Table 2). According to the XPS analysis, the fraction of Cu^2+^ cations is about 78% and 52% for Cu/TiO_2_ and Cu/TiO_2_ 700, respectively (Figure S3a). However, it should be stressed that the identification of Cu^1+^ and Cu^0^ states by analyzing the Cu*2p* spectrum is difficult, as the Cu*2p_3/2_* binding energies of Cu^1+^ and Cu^0^ states are similar [36]. Nevertheless, the Auger parameter α, which is equal to the sum of the Cu*2p_3/2_* peak binding energy and the Cu *LMM* peak kinetic energy, can be used for the identification of Cu^1+^ and Cu^0^ states. Unfortunately, the surface concentration of copper cations did not allow us to measure the Cu *LMM* peak kinetic energy and identify the cation distribution. It should also be noted that the [Cu]/[Ti] surface ratios are slightly different for Cu/TiO_2_ and Cu/TiO_2_ 700. The observed deviation is explained by the surface sensitivity of XPS, which is affected by different size distributions of copper nanoparticles, the presence of large Cu/CuO_x_ particles (in the case of Cu/TiO_2_), and a decrease in the surface area of TiO_2_. Although the presence of CuO_x_ is beneficial for light absorption in the visible region, due to the agglomeration of copper nanoparticles and the presence of large Cu/CuO_x_ particles in the sample Cu/TiO_2_ (Figure S2a), Cu/TiO_2_ cannot work as efficiently as the Cu/TiO_2_ 700 photocatalyst. The study of catalysts after irradiation by LED-400 nm and LED-450 nm does not reveal any significant changes in Cu/TiO_2_ 700 (Figure S3b) when the [Cu]/[Ti] surface ratio decreases in the case of Cu/TiO_2_. Moreover, the XPS analysis of Cu/TiO_2_ 700 after 120 h of irradiation by LED-400 nm clearly demonstrates its stability.

Based on the kinetic results and the photocatalyst characterization, we can speculate that the product distribution of the CO_2_ reduction process depends on the nature of the cocatalyst and the optical absorption edge of the photocatalyst. Provided that there is enough energy of photons for the generation of a relatively high number of electrons, electrons can be collected in a cocatalyst and participate in the multi-electron process of CO_2_ reduction to CH_4_ (Equation (3)) [37]. The photon energy required could be reduced by depositing a cocatalyst, which can absorb light with a longer wavelength, and/or by changing the adsorption edge of TiO_2_. The combination of these two approaches makes it possible to obtain a photocatalyst exhibiting high activity with respect to methane under irradiation with 425 nm wavelength. The formation rate of CH_4_ over the Cu/TiO_2_ 700 sample achieves 1.2 ± 0.1 μmol h^−1^ g^−1^ and an overall CO_2_ reduction rate of 11 ± 1 μmol h^−1^ g^−1^ (for a LED-425 nm). A possible reason for the low CH_4_ formation rate over Cu/TiO_2_ and Cu/TiO_2_ 700 when using LEDs with a wavelength of 365 and 380 nm is the different electron surface densities in the photocatalyst. Since TiO_2_ has strong adsorption in the UV region, it can effectively generate electron–holes pairs under LED-365 and LED-380. It has been shown that CO_2_ reduction on the TiO_2_ surface predominantly led to CO formation [38]. Under visible light irradiation, copper oxides absorb light to a much greater extent than TiO_2_, therefore, the main generation of charge carriers occurs in cocatalyst particles (Cu/CuO_x_), and the most of the electrons are located in the metal, which leads to an increase in the rate of methane formation [39].

The photocatalytic activity of Cu/TiO_2_ 700 at 425 nm is caused by several factors. Firstly, the absorption edge of the sample is around 400 nm, but since the light absorption of TiO_2_ 700 decreases gradually from 330 to 440 nm, the photocatalyst is still able to absorb light irradiation beyond 400 nm, although to a lesser extent. Additionally, since LEDs have non-monochromatic radiation, LED-425 emits light with a wavelength of both more and less than 425 nm, as shown in Figure 1a. Finally, the presence of CuO_x_ greatly promotes the adsorption of visible light, as stated in the Introduction section. We believe that the observed behavior of Cu/TiO_2_ 700 will promote the use of Cu-TiO_2_-based catalysts for the conversion of CO_2_ under visible light.

Additionally, it should be noted that the fraction of Cu^2+^ and Cu^0^/Cu^1+^ cations varies after the photocatalytic reaction for both the Cu/TiO_2_ and Cu/TiO_2_ 700 catalysts (Table 2). This behavior indicates that copper cations play key roles in the reduction of CO_2_ and could influence product distribution. The photogenerated electrons could reduce the copper oxides according to the following equations [40]:

Cu^2+^ + e^−^ = Cu^+^, E^0^(Cu^2+^/Cu^1+^) = 0.16 V (7)

Cu^2+^ + 2e^−^ = Cu^0^, E^0^(Cu^2+^/Cu^0^) = 0.34 V (8)

Cu^+^ + e^−^ = Cu^0^, E^0^(Cu^1+^/Cu^0^) = 0.52 V (9)

while the reduced states could be oxidized by the free oxygen anion radicals generated due to the reaction of O_2_ with e^−^ (Equation (10)) or by singlet oxygen, which is the product of a reaction between O_2_^−^ and h^+^ (Equation (11)) [41].

O_2_ + e^−^ = O_2_^−^ (10)

O_2_^−^ + h^+^ = ^1^O_2_ (11)

Therefore, ternary heterostructures, including Cu, CuO_x_, and TiO_2_, can be formed during the photocatalytic reaction. Both CuO_x_ and TiO_2_ compounds adsorb light irradiation and generate electron–hole pairs. CuO (p-type) and TiO_2_ (n-type) form a p–n heterojunction, and the electron transfer from TiO_2_ to CuO suppresses the charge recombination [42,43,44]. The charge separation between TiO_2_ and Cu_2_O is realized according to the Z-scheme, according to which electrons from the conduction band (CB) of TiO_2_ recombine with holes from the valence band of Cu_2_O [45]. The CB of Cu_2_O is −1.55 eV [46], which is much lower than the potential of CO_2_ reduction to CO and CH_4_ in the presence of water (Equations (2) and (3)). Therefore, the remaining electrons in Cu_2_O can easily react with CO_2_ to produce CO or CH_4_. At the same time, holes in TiO_2_ react with water vapor. The working function of Cu is higher than that of TiO_2_ and CuO_x_; therefore, the presence of metallic Cu promotes electron transfer to metal particles from TiO_2_, as well as from CuO_x_, thus improving the charge separation and the lifetime of e^-^ and h^+^ [47,48,49,50]. Depending on the position of copper and which contacts are present in the catalyst (Cu-TiO_2_, Cu-CuO_x_, or both of them), various mechanisms for the transfer of photogenerated charge carriers can be realized. This is a difficult task and requires another detailed study, for example, in situ/operando X-ray absorption spectroscopy (XAS) study of Cu *K*-edge during the photoreduction of CO_2_. It should be noted that XAS is more sensitive to the oxidation states of copper cations than XPS and allows the Cu^2+^, Cu^1+^, and Cu^0^ states to be easily distinguished [51].

### 3.3. Stability Test

**Figure 7:**
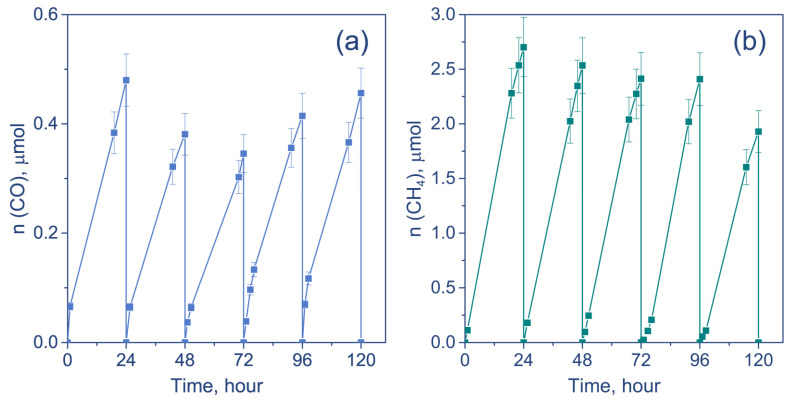
The amounts of (**a**) CO and (**b**) CH_4_ formed during the stability test. Conditions: m(cat.) = 30 mg, P_0_(CO_2_) = 1 atm, t (1 run) = 24 h, λ = 400 nm.

To study the catalytic stability of the most active catalyst (Cu/TiO_2_ 700), stability tests were carried out. The activity of Cu/TiO_2_ 700 catalyst was tested during five 24 h runs under irradiation of LED-400 nm (Figure 7). Between the runs, light irradiation was turned off, and the reactor was purged with CO_2_ for 1 h.

**Figure 8:**
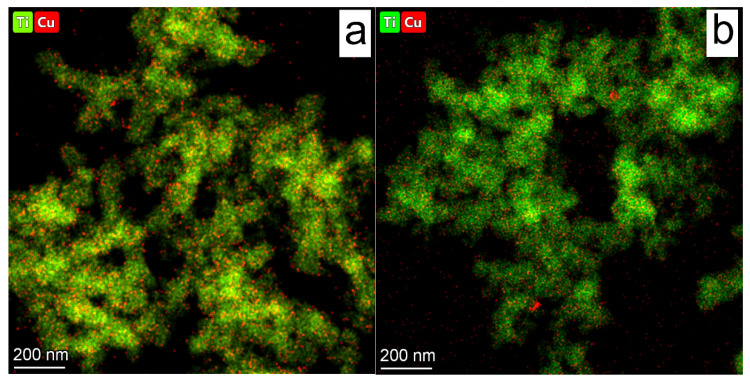
EDX mapping of (**a**) fresh Cu/TiO_2_ 700 sample and (**b**) after irradiation for 120 h or 5 runs under LED-400 nm.

The CO formation rate slightly decreases during the second and third runs and then begins to grow; the rate of CH_4_ formation stays constant throughout the first four runs and then decreases. In general, the photocatalyst exhibits high stability during 120 h of CO_2_ reduction. The total amount of CH_4_ is 400 µmol g^−1^ and, for CO, is 70 µmol g^−1^ after 120 h of irradiation. The observed decrease in photocatalytic activity could be due to the decrease in the number of active centers. It was mentioned above that eight electrons are necessary to convert CO_2_ to CH_4_, whereas for CO production, two electrons are needed. Thus, the decrease in the number of heterojunctions could lead to a shift in the rate of CO_2_ reduction to CO. It is likely that a decrease in the number of active centers is due to the increased size of copper nanoparticles. It is known that copper oxides could undergo photocorrosion caused by photoelectron reduction [52]. It has been previously observed that the reduction of transition metal oxides to the metallic phase could lead to the migration of metallic nanoparticles over the surface and the formation of metallic agglomerates [53,54]. The formation of agglomerates could lead to a decrease in the number of active sites and, thus, a decrease in photocatalytic activity. We believe that the calcination of TiO_2_ leads to the formation of sites where the copper nanoparticle could be fixed, so as not to allow them to migrate over the surface and form the agglomerates. Indeed, as it was mentioned above, the EDX mapping shows that, in the case of Cu/TiO_2_ catalyst, the agglomerates of copper nanoparticles are found, as well as large copper particles of 100–200 nm (Figure S2a). At the same time, the EDX analysis does not reveal any copper particles of 10–100 nm in the case of Cu/TiO_2_ 700 catalysts (Figure 8a). The stabilization of nanoparticles, which prevents the aggregation of Cu/CuO_x_ nanoparticles on the support, is the key to increasing the long-term stability of photocatalysts. Additionally, the EDX analysis of Cu/TiO_2_ catalyst exposed under LED-450 nm for 24 h reveals an increase in the number of copper particles with sizes of 100–200 nm (Figure S2b,c). To investigate its long-term stability, the Cu/TiO_2_ 700 catalyst was studied using EDX after the fifth run of CO_2_ reduction under irradiation of LED-400 nm (Figure 8b). One can see two regions enriched with Cu. The size of these agglomerates is about 20 nm, and that is much lower than Cu particles observed after 24 h of irradiation of Cu/TiO_2_ (Figure S2c). Additionally, the distribution of oxygen (Figure S4a,b) in Cu/TiO_2_ 700 after five runs indicates the absence of an area enriched with O at the place of Cu atom agglomeration. It can be concluded that this region, enriched in Cu atoms, mainly consists of metallic copper. Such metallic particles can be formed by the migration of metallic nanoparticles due to the reduction of copper oxides, as mentioned above. Therefore, the decrease in CH_4_ production clearly observed on the fifth run could be caused by the agglomeration of Cu/CuO_x_ particles, thus decreasing contact between cocatalyst and TiO_2_ particles and reducing the lifetime of electrons. Additionally, the reduction of CuO_x_ to Cu decreases the visible light absorption by photocatalyst.

The results reveal that the calcination of TiO_2_ at 700 °C facilitates the deposition of well-dispersed cocatalyst particles and prevents Cu/CuO_x_ aggregation during even 100 h of the photocatalytic reaction. This effect promotes the CH_4_ formation rate rather than the formation of CO. Additionally, the presence of copper oxides provides increased adsorption of visible light by the photocatalyst, which is vital for carrying out the multielectron process of CO_2_ reduction to CH_4_ upon irradiation in the visible region.

## 4. Conclusions

In this study, the influence of a cocatalyst and thermal activation of TiO_2_-based photocatalysts on the CO_2_ reduction process was investigated. Platinized photocatalysts were shown to be more active in relation to the CH_4_ formation under UV light irradiation, while Cu-containing ones demonstrated superior activity in CH_4_ production upon irradiation with visible light. The maximum overall CO_2_ reduction rate under UV irradiation was 2.8 µmol h^−1^ g^−1^, which was achieved over TiO_2_ calcinated at 700 °C and modified with Pt. The Cu/TiO_2_ showed an activity rate of 2.6 µmol h^−1^ g^−1^. The highest CO_2_ reduction rate was 37 µmol h^−1^ g^−1^, achieved over Cu/TiO_2_ under light irradiation with a maximum intensity of 400 nm. At 425 nm, the Cu/TiO_2_ 700 demonstrated a maximum CO_2_ reduction rate of 11 µmol h^−1^ g^−1^. Both rates are high, compared with the published data on CO_2_ reduction over different photocatalysts [3,55,56]. Under purely visible light irradiation (LED-425 nm), AQE and S(CH_4_) for Cu/TiO_2_ 700 photocatalyst were achieved by 0.23% and 90%, respectively. Therefore, the replacement of platinum with copper is an effective method for the synthesis of a visible active photocatalyst for CO_2_ reduction, provided that calcined TiO_2_ (700 °C) is used.

The stability of Cu/TiO_2_ 700 was tested during 120 h of reaction under LED-400 nm. It was shown that the cocatalyst particles began to aggregate during experiments, but this process proceeded much more slowly on the surface of calcined TiO_2_. Platinum was in the metallic state on the surface of both TiO_2_ and TiO_2_ 700 samples. Copper was present in various states, and the Cu/TiO_2_ 700 sample contained less Cu^2+^ than Cu/TiO_2_, but this sample had a uniform distribution of cocatalyst, which is beneficial for the CH_4_ production under irradiation of visible light. This photocatalyst demonstrated fairly good stability over 120 h of CO_2_ reduction. A slight decrease in the CH_4_ formation rate is probably associated with the beginning enlargement of Cu/CuO_x_ particles and the reduction of copper oxides to metal particles, which reduces light absorption in the visible region. 

## Figures and Tables

**Table 1:** Relative atomic concentrations of elements in the surface area of the catalysts under study and Pt*4f_7/2_* and O*1s* binding energies.

Sample	[Pt]/[Ti]	[O]/[Ti]	Binding Energy, eV	
			Pt*4f_7/2_*	O*1s*
Pt/TiO_2_	0.009	2.50	70.9 (Pt^0^)	530.3
Pt/TiO_2_ 700	0.020	2.53	70.9 (Pt^0^)	530.3
Pt/TiO_2_ (LED-400 nm)	0.009	2.51	70.8 (Pt^0^)	530.3
Pt/TiO_2_ 700 (LED-400 nm)	0.016	2.56	70.9 (Pt^0^)	530.3

**Table 2:** Atomic ratios of elements in the surface layer of samples Cu/TiO_2_ and Cu/TiO_2_ 700 before and after photocatalytic reaction. In parentheses is the diode that was used to carry out the reaction in the case of samples tested.

Sample	[Cu]/[Ti] ^1^	[Cu*]/[Ti] ^2^	[O]/[Ti]	Cu*, % ^2^	Cu^2+^, %
Cu/TiO_2_	0.28	0.06	2.64	22	78
Cu/TiO_2_ 700	0.20	0.10	2.66	48	52
Cu/TiO_2_ (400 nm)	0.26	0.05	2.63	18	82
Cu/TiO_2_ (450 nm)	0.22	0.05	2.61	25	75
Cu/TiO_2_ 700 (400 nm)	0.20	0.08	2.71	41	59
Cu/TiO_2_ 700 (450 nm)	0.26	0.08	2.66	32	68
Cu/TiO_2_ 700 (400 nm, 120 h of reaction)	0.19	0.11	2.63	57	43

^1^ Cu—total Cu in all states (Cu^0^, Cu^1+^, and Cu^2+^); ^2^ Cu*—Cu in the states Cu^0^ and Cu^1+^.

## Data Availability

The data presented in this study are available on request from the corresponding author.

## Supplementary Materials

The following supporting information can be downloaded at: https://www.mdpi.com/article/10.3390/nano12091584/s1, Figure S1: Pt*4f* core-level spectra of the fresh (a) Pt-TiO_2_ photocatalysts and after irradiation (b) under LED-400 nm. The spectra are normalized to the integral intensity of the corresponding Ti*2p* core-level spectra. Figure S2: EDX mapping (in HAADF STEM mode) of the fresh Cu/TiO_2_ (a) catalyst and after irradiation (b,c) for 24 h under LED-450 nm. Figure S3: Cu*2p* core-level spectra of the fresh (a) Cu-TiO_2_ photocatalysts and after irradiation (b) under LED-400 nm. Figure S4: EDX mapping (in HAADF STEM mode) of the Cu/TiO_2_ 700 catalyst after irradiation for 120 h under LED-400 nm (Cu (a), O (b)).
