# Supplementary material for: Broadening the Action Spectrum of TiO2-Based Photocatalysts to Visible Region by Substituting Platinum with Copper
Source: Nanomaterials (Basel). 2022 May 7;12(9):1584. doi: 10.3390/nano12091584 (PMC9105519; doi:10.3390/nano12091584)
Supplement: Supplementary file 1 [file nanomaterials-12-01584-s001.zip › nanomaterials-1707700-supplementary.pdf]

Supplementary Materials

Broadening the Action Spectrum of TiO_2-Based Photocatalysts to Visible Region by Substituting Platinum with Copper

Andrey A. Saraev, Anna Yu. Kurenkova, Evgeny Yu. Gerasimov and Ekaterina A. Kozlova *

Federal Research Center, Boreskov Institute of Catalysis SB RAS, Lavrentieva Ave. 5, 630090 Novosibirsk, Russia; asaraev@catalysis.ru (A.A.S.); kurenkova@catalysis.ru (A.Y.K.); gerasimov@catalysis.ru (E.Y.G.)

* Correspondence: kozlova@catalysis.ru

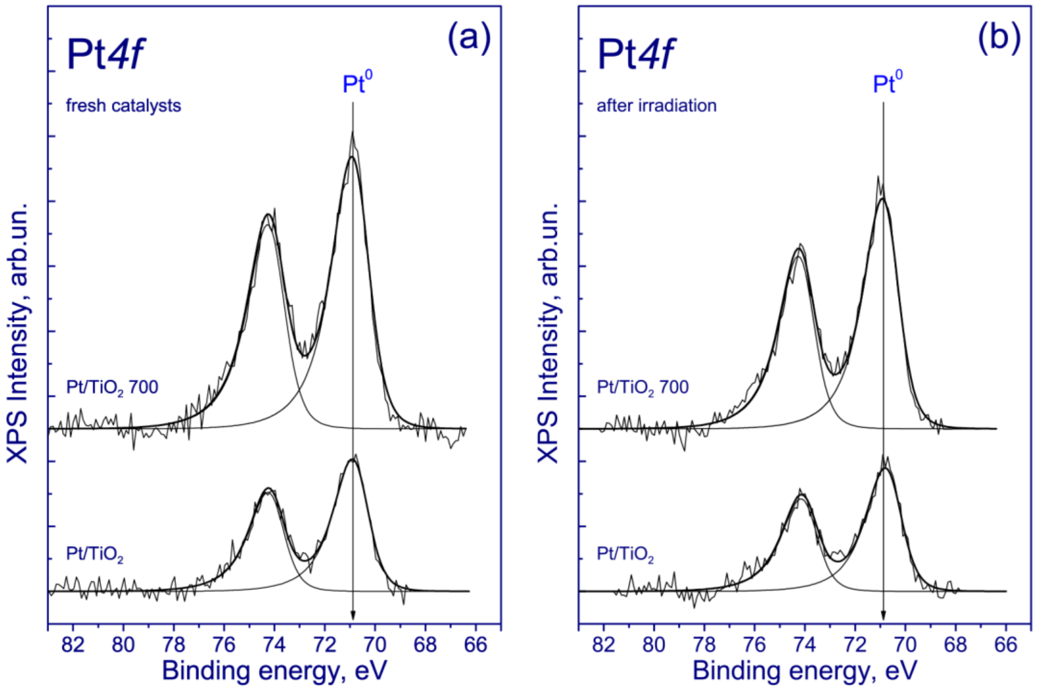

Figure S1. Pt4*f* core-level spectra of the fresh (**a**) Pt-TiO_2 photocatalysts and after irradiation (**b**) under LED-400 nm. The spectra are normalized to the integral intensity of the corresponding Ti2*p* core-level spectra.

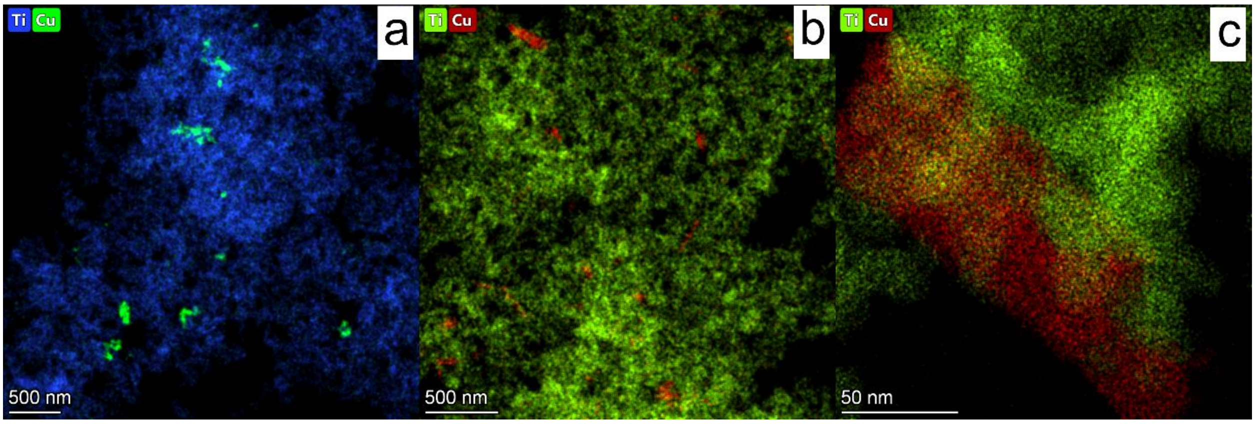

Figure S2. EDX mapping (in HAADF STEM mode) of the fresh Cu/TiO_2 (**a**) catalyst and after irradiation (**b**,**c**) for 24 h under LED-450 nm. It is clearly seen that the increase in the quantity of copper particle of 100–200 nm after irradiation.

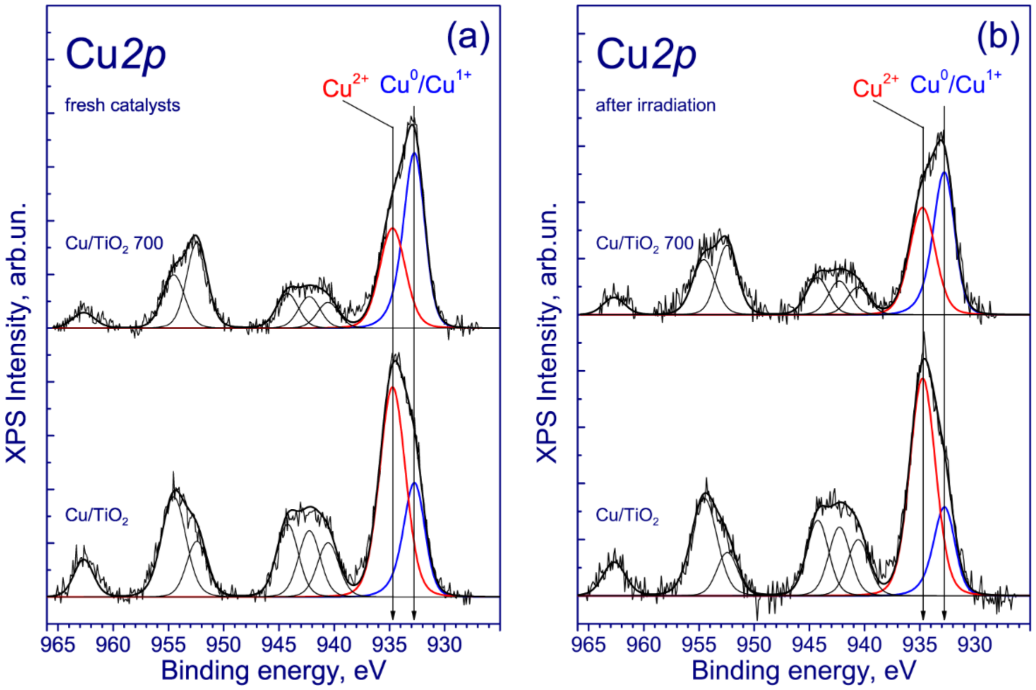

Figure S3. Cu2*p* core-level spectra of the fresh (**a**) Cu-TiO_2 photocatalysts and after irradiation (**b**) under LED-400 nm. The spectra are normalized to the integral intensity of the corresponding Ti2*p* core-level spectra.

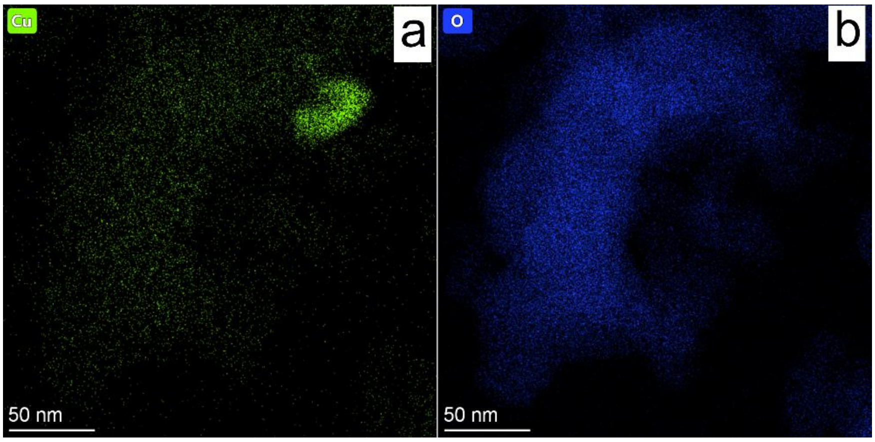

Figure S4. EDX mapping (in HAADF STEM mode) of the Cu/TiO_2 700 catalyst after irradiation for 120 h under LED-400 nm (Cu (**a**), O (**b**)). It is clearly seen that the formation of Cu metallic particles takes place.
